# Evaluating femoral head collapse risk post-fixation removal: a finite element analysis

**DOI:** 10.3389/fbioe.2025.1441026

**Published:** 2025-03-06

**Authors:** Xishan Li, Xiang Zhou, Jie Yang, Kai Oliver Böker, Arndt F. Schilling, Wolfgang Lehmann

**Affiliations:** ^1^ Department of Trauma Surgery, Orthopedics and Plastic Surgery, University Medical Center Göttingen, Göttingen, Germany; ^2^ Department of Articular and Traumatic Orthopedic Surgery, Fourth People’s Hospital of Guiyang, Guiyang, Guizhou, China

**Keywords:** finite element analysis, femoral head collapse, screw hole configuration, internal fixation removal, femoral neck fracture

## Abstract

**Background:**

Femoral neck fractures are prevalent in orthopedic injuries, often leading to complications such as nonunion and osteonecrosis of the femoral head (ONFH). Studies indicate that after healing and removal of internal fixation devices, some patients develop ONFH, while others experience osteosclerosis around the screw holes due to prolonged fixation, increasing ONFH risk. Despite such observations, biomechanical studies on this phenomenon are limited. This study assesses the risk of femoral head collapse post-internal fixation device removal and investigates the biomechanical effects of bone grafting at screw removal sites.

**Methods:**

Using CT data, femoral anatomy was reconstructed. For control, the femoral head’s collapse area was identified. Experimental models, divided into those with and without bone grafts in screw holes, incorporated three fixation techniques, namely, triple cannulated screws (3CS), dynamic hip screws with cannulated screws (DHS+CS), and the femoral neck system (FNS), further subclassified into normal and sclerotic screw-hole models. Stress distribution, stress values, stress index, and strain range were assessed.

**Results:**

In both models, DHS+CS showed the highest stress in the overall model, while 3CS had the highest stress in the collapse area. The 3CS configuration also resulted in the largest strain range, which was observed in the central pillar of normal screw-hole models and the lateral pillar of sclerotic screw-hole models. The bone graft models exhibited lower peak, average stress, and strain values than the normal and sclerotic screw-hole models.

**Conclusion:**

The FNS screw hole demonstrates a relatively lower mechanical risk of femoral head collapse. In contrast, sclerotic screw holes increase this risk, while bone grafting may improve the biomechanical behavior after fixation removal, potentially reducing the likelihood of femoral head collapse.

## 1 Introduction

Femoral neck fractures are a common type of joint trauma, accounting for nearly 50% of proximal femur fractures (Wang et al., 2019). In Germany, the incidence has increased by 23% over the past decade (Szymski et al., 2023). Furthermore, mortality rates associated with femoral neck fractures remain elevated, ranging at approximately 8.4%–36% within the first year following surgery (Abrahamsen et al., 2009; Mundi et al., 2014). Due to advancements in internal fixation techniques, the healing rate for femoral neck fractures has surpassed 90% (Bhandari and Swiontkowski, 2017). At present, the predominant surgical interventions for femoral neck fractures in patients include triple cannulated screws (3CS), dynamic hip screws with cannulated screws (DHS+CS), and the femoral neck system (FNS) (Huang et al., 2010; Yang et al., 2013; Niemann et al., 2023). Following the healing of femoral neck fractures, the removal of screws is often necessitated due to functional impairment, local irritation, or subjective discomfort experienced by the patient (Hanson et al., 2008; Zielinski et al., 2015). However, some studies suggest that screw removal after femoral neck fracture healing is linked to an increased incidence of osteonecrosis of the femoral head (ONFH). In these cases, nail removal significantly raises the risk of avascular necrosis of the femoral head. The incidence of ONFH in the implant removal group was 4.167 times higher than that in the group without implant removal, and this risk increased to 26.0 times in elderly patients who underwent implant removal (Ai et al., 2013; Li et al., 2018). The femoral neck may be unable to fully withstand these forces, leading to a disruption in the biomechanical stress and load-bearing function of its trabeculae. This disruption can result in microfractures or even re-fractures of the femoral neck (Shah et al., 2015). Additionally, Kim et al. identified a high incidence of hidden ONFH, with MRI detecting 13 cases in 58 patients after fixation removal. Four patients experienced femoral head collapse, one of whom required hip replacement surgery, highlighting the need to carefully select fixation methods that minimize this risk (Kim et al., 2022). Furthermore, the screw hole is the most common and critical contributor that increases the femoral stress and the risk of re-fractures after removing the implant (Zhou et al., 2019), while placing resorbable fillers in bone defects after hardware removal could reduce the risk of re-fractures (Alford et al., 2007). Conversely, a recent study indicated that prolonged retention of the internal fixation implant following surgery for femoral neck fracture can result in osteosclerosis around the screw holes in the femoral head. The presence of sclerotic bone may contribute to the development of ONFH (Liu et al., 2022b). Therefore, the need for screw removal after the union of a femoral neck fracture remains controversial. ONFH, which typically affects the weight-bearing region, is a destructive disease, leading to femoral head collapse. This condition often necessitates total hip arthroplasty. In young patients, the increased incidence of ONFH secondary to femoral neck fracture might be associated with increased daily activity (Goudie et al., 2018). A meta-analysis encompassing 747 patients with FNF, the majority of whom were treated with cannulated screws, revealed that the removal of internal fixation is associated with an increased incidence of ONFH in patients with healed fractures (Jiang et al., 2023). However, the mechanical strength of the femoral head associated with different screw-hole configurations after the removal of internal fixation is also not well-understood, and the biomechanical changes of the screw holes after utilizing the resorbable fillers remain unclear. Thus, it is necessary to investigate the biomechanical behavior of the screw hole in the femoral head after the removal of internal fixation.

We hypothesize that different screw-hole configurations may influence the mechanical strength of the femoral head after internal fixation removal. This study aimed to compare the risk of femoral head collapse by analyzing the biomechanical behavior of varying screw-hole structures and assessing the influence of the sclerotic zone in the femoral head. Additionally, we investigated the biomechanical changes in the femoral head after bone grafting in the screw hole to provide a reference for predicting the risk of femoral head collapse after removing internal fixation.

## 2 Materials and methods

### 2.1 Materials

Data: a 50-year-old male patient presented without any historical incidence of femoral trauma. The imaging assessments revealed an absence of tumors, deformities, and fractures. It was provided by the Department of Radiology at the University Medical Center Göttingen.

Computer workstation: processor: Intel(R) Core (TM) i9-10900 K CPU @ 3.70 GHz; installed RAM: 64.0 GB; graphics card: Nvidia Quadro RTX 4000 (8.0 GB).

Software: 3D reconstruction: 3D Slicer 5.0.2 (https://www.slicer.org); 3-Matic 9.0 (Materialise, Leuven, Belgium); Geomagic Wrap 2021 (3D Systems, United States).

Model assembly: Solid Works 2018 (Dassault Systèmes, France).

FE-analysis software: ANSYS 2021R2 (ANSYS, United States).

### 2.2 Methods

#### 2.2.1 FE-model designs and groups

**FIGURE 1:**
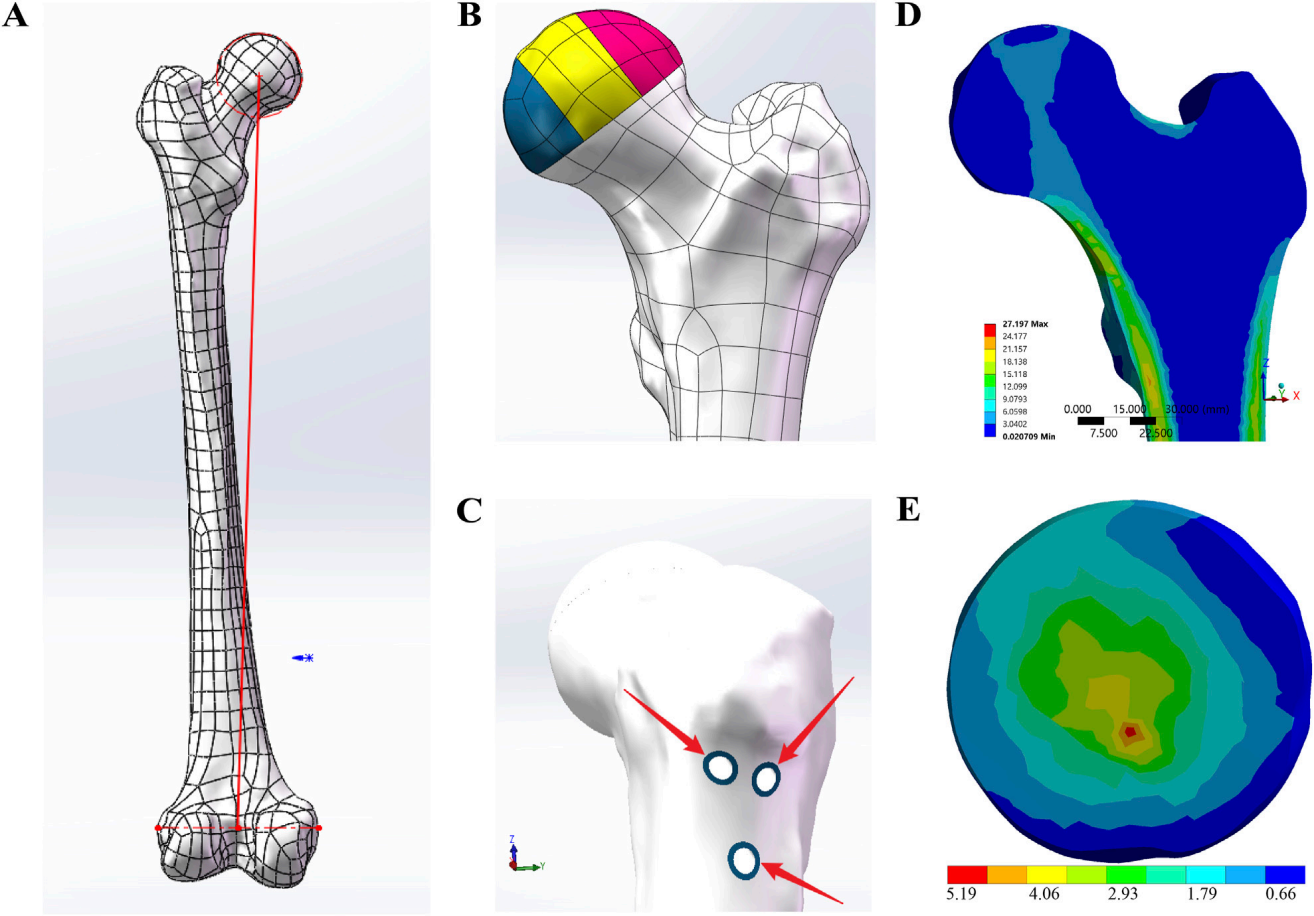
FE-model designs. **(A)** The red line represents the lower limb mechanical axis, which was used as the coordinate system to define the loading direction in ANSYS. **(B)** According to the three-pillar classification of ONFH, the collapsed necrotic region of the femoral head was divided into the lateral pillar (colored pink), the central pillar (colored yellow), and the medial pillar (colored blue). **(C)** The blue sclerotic zone surrounding the screw holes was determined to be 1.3533 mm. **(D)** Distribution of principal stress from the apex of the femoral head to the calcar in the sagittal view. **(E)** Stress distribution in the collapse area of the control group.

The healthy femur model was constructed using segmentation and modeling tools in 3D Slicer based on CT scan data. The experimental solid models were created by reverse-engineering the initial femur model into a CAD format using Geomagic Wrap, followed by further modifications in SolidWorks. The coordinates of the lower limb’s mechanical axis were then established (Cooke and Sled, 2009) (Figure 1A). Subsequently, the distal 24 cm of the femur was removed, leaving only the proximal portion for the experiment. The model was then imported into ANSYS for meshing and subsequently into 3-matic. Using established formulas from the previous literature (Wang et al., 2017), the bone density was calculated from CT’s Hounsfield units (HUs), and then, corresponding material properties were applied.

##### 2.2.1.1 Control group

Based on the three-pillar classification of ONFH (Wen et al., 2020), the collapsed necrotic area of the femoral head was divided into the lateral, central, and medial pillars in a ratio of 3:4:3 to facilitate observation and comparison of the collapse (Figure 1B).

##### 2.2.1.2 Experimental group

Based on data provided by the previous literature reports (Fan et al., 2021; Wang et al., 2021), solid models of the CS, DHS, and FNS were created in SolidWorks. Subsequently, adhering to standard surgical fixation techniques, three distinct internal fixation methods—3CS, DHS+CS, and FNS—were assembled onto the anatomical femur model. Following this, within SolidWorks, various internal fixations were subtracted from the model, thereby leaving screw holes on the femur model. According to the research by Liu et al. (2022a), the average thickness of the sclerotic zone surrounding femoral screw tracks was determined to be 1.3533 mm. Building upon the screw-hole models, a U-shaped entity with a thickness of 1.3533 mm was precisely modeled along the screw holes, as shown in Figure 1C. Within the previously mentioned models, a healthy cancellous bone graft, precisely matching the screw tracks, was used to fill the collapsed necrotic region of the femoral head, thereby creating the bone graft screw-hole models. Finally, the same three-pillar observation region for ONFH was established with reference to the control group model.

#### 2.2.2 *In silico* FE-model settings and solutions

##### 2.2.2.1 Material properties

Based on Zhan et al. (2024), to construct the femoral model, we first utilized HU values obtained from CT scans to estimate bone density (ρ) using an established correlation.

$$\rho \left(g/cm^{3}\;\right)=0.000968\times HU+0.5.$$

Subsequently, the elastic modulus (E) and Poisson’s ratio (ν) were calculated based on the derived density using the following relationships:

If $\rho \leq 1.2\;\frac{g}{cm^{3}},$

$$E=2014\times \rho^{2.5}\;\left(MPa\right),\nu =0.2.$$

If $\rho \;>\;1.2\;\frac{g}{cm^{3}},$

$$E=1763\times \rho^{3.2}\;\left(MPa\right),\nu =0.32.$$

In accordance with recent studies on sclerotic bone surrounding femoral head screw paths (Liu et al., 2022b), the sclerotic cortical bone was assigned an elastic modulus of 13,300 MPa and a Poisson’s ratio of 0.3, while the sclerotic cancellous bone was assigned an elastic modulus of 240.07 MPa and a Poisson’s ratio of 0.2. The material properties of the bone graft were modeled after healthy cancellous bone, with an elastic modulus of 121 MPa and a Poisson’s ratio of 0.3 (Hou and Zhu, 2006).

##### 2.2.2.2 Contact conditions

The regions between the three pillars for ONFH, between the pillars and the distal femur, and between the sclerotic zone and the surrounding normal bone were meshed with shared nodes. The contact between the bone graft and either the normal bone or the sclerotic zone is set as bonded.

##### 2.2.2.3 Boundary and loading conditions

The surface of the distal femur was immobilized in all directions. A load of 2,100 N was applied in the direction of the lower extremity mechanical axis at the weight-bearing area of the femoral head.

#### 2.2.3 Evaluation criteria

The collapsed area, which typically occurs in the femoral head and involves the three pillars, was the primary focus of observation. Therefore, the stress distribution and stress index of the collapsed area, as well as the strain range of the three pillars, were analyzed.

Additionally, the peak stress and average stress in the overall model; the collapsed area; and the lateral, central, and medial pillars were examined.

#### 2.2.4 Model validation

The FE models were meshed using 10-node tetrahedral elements, resulting in fine meshes comprising 417,561 elements and 588,262 nodes. Sensitivity analyses indicated that increasing the element density did not enhance the model’s prediction accuracy but did elevate the computational load. Convergence was confirmed when results varied by less than 5% over three consecutive steps of sufficient mesh refinement. The final element size determined through this process was applied uniformly to all FE models. To ensure overall mesh quality, the von Mises stress and surface displacement errors were calculated for each FE model individually (Oefner et al., 2021).

The characteristics of the principal stress transfer path are critical for evaluating femoral biomechanical indices. Figure 1D illustrates the distribution of principal stress from the apex of the femoral head to the calcar in the cross-sectional view. The simulation results align with findings from previous studies (Xiao et al., 2015; Zhou et al., 2015). Furthermore, the shape and location of the biomechanical transfer path correspond closely with the distribution of primary compressive trabeculae (Zhang et al., 2023).

Additionally, FE-analyses in this study were also assessed based on stiffness, maximum von Mises stress, maximum von Mises strain, and 8-node von Mises stress and compared with relevant findings from previous *in vitro* or finite element analysis studies (Papini et al., 2007; Yosibash et al., 2007; Fu et al., 2012; San Antonio et al., 2012; Chen et al., 2019; Jian-Qiao Peng et al., 2020). As shown in Tables 1–4, the model developed in this study demonstrates good consistency and reliability with previous reports. Therefore, we hypothesize that the FE results can accurately reflect the physical status of the femur and can be used to analyze the impact of different screw track structures on femoral head collapse.

**TABLE 1:** Model validation: stiffness (kN/mm).

Literature	Stiffness
Papini et al. (2007)	0.757 ± 0.264
Chen et al. (2019)	0.54
Own	0.5856

**TABLE 2:** Model validation: maximum von Mises stress (MPa).

Literature	Maximum von Mises stress
Fu et al. (2012)	22
San Antonio et al. (2012) (1)	17.95
San Antonio et al. (2012) (2)	17.49
San Antonio et al. (2012) (3)	18.05
Own	17.969

**TABLE 3:** Model validation: maximum von Mises strain.

Literature	Maximum von Mises strain
San Antonio et al. (2012) (1)	0.002
San Antonio et al. (2012) (2)	0.0022
San Antonio et al. (2012) (3)	0.0023
Yosibash et al. (2007)	0.00153
Own	0.00229

**TABLE 4:** Model validation: eight nodes von Mises stress (MPa).

Matthew 2020	Node 1	Node 2	Node 3	Node 4	Node 5	Node 6	Node 7	Node 8
FE model	1.749	1.008	1.561	0.476	2.954	2.089	1.103	0.694
Cadaver	2.081	0.84	2.464	0.256	3.261	2.792	1.369	0.219
Own	2.053	0.978	1.911	0.333	3.102	2.251	1.15	0.341

## 3 Result

### 3.1 Evaluation of equivalent stress and stress distributions

The peak and average stress values for the health, normal screw hole, and bone graft models are presented in Table 5. In both the normal screw hole and bone graft models, the peak and average stresses in the overall model, collapse area, lateral pillar, central pillar, and medial pillar were higher than those in the healthy model. However, the bone graft models exhibited lower stresses in all areas compared to the normal screw-hole models. In the overall model, the DHS+CS screw-hole configuration showed the highest peak and average stress values in both the normal screw hole and bone graft groups. In the normal screw-hole group, the peak stress of DHS+CS was 50.54 MPa, and the average stress was 4.38 MPa. In the bone graft group, the peak and average stresses were 46.32 MPa and 4.33 MPa, respectively. In the collapse area, the 3CS screw-hole configuration had the highest peak and average stress values for both the normal screw hole and bone graft groups. In the normal screw-hole group, the peak stress of 3CS was 12.22 MPa, and the average stress was 1.72 MPa. In the bone graft group, the peak stress was 10.23 MPa, and the average stress was 1.6 MPa. In the lateral, central, and medial pillars, the highest peak and average stress values were observed in the central pillar for both the normal screw hole and bone graft models. In contrast, the healthy model showed the highest peak and average stresses in the lateral pillar. Regarding the stress index in the collapse area, only the 3CS screw-hole configuration exhibited a value greater than 0.1 in both the normal screw hole and bone graft groups.

**TABLE 5:** Stress in healthy, normal screw hole, and bone graft models.

Model	Peak (average) stress (Mpa)					Stress index of collapse area^b^
	Overall model	Collapse area^a^	Lateral pillar	Central pillar	Medial pillar	
Healthy model	27.2 (4.16)	5.19 (1.5)	**5.19 (1.74)**	5.12 (1.73)	2.42 (0.7)	0.031–0.052
3CS	27.45 (4.23)	**12.22 (1.72)**	5.83 (1.85)	**12.22 (2.07)**	3.67 (0.81)	**0.073–0.123**
DHS+CS	**50.54 (4.38)**	7.89 (1.72)	5.51 (1.85)	**7.89 (1.96)**	4.36 (1.05)	0.079–0.047
FNS	46.66 (4.36)	7.63 (1.7)	5.5 (1.83)	**7.63 (2)**	4.51 (0.91)	0.077–0.045
3CS (bone graft)	28.44 (4.17)	**10.23 (1.6)**	5.78 (1.82)	**10.23 (1.97)**	3.32 (0.76)	**0.061–0.103**
DHS+CS (bone graft)	**46.32 (4.33)**	6.82 (1.58)	5.53 (1.8)	**6.82 (1.86)**	3.89 (0.9)	0.041–0.069
FNS (bone graft)	44.74 (4.33)	7.26 (1.58)	5.55 (1.8)	**7.26 (1.93)**	2.97 (0.86)	0.043–0.073

^a^ The collapse area is the collapsed necrotic region that typically occurs in the femoral head, involving the lateral, central, and medial pillars.

^b^ Stress index = effective stress/yield strength. Microfractures form in the collapse area when the stress index is >0.1. In the Overall model, the peak (average) stress was highest in the DHS+CS model, regardless of whether the normal screw hole models or the bone graft models was used, with values of 50.54 (4.38) and 46.32 (4.33), respectively. In the Collapse area, the peak (average) stress was highest in the 3CS model, for both the normal screw hole models and the bone graft models, with values of 12.22 (1.72) and 10.23 (1.6), respectively. For the three-column regions (Lateral, Central, and Medial pillars), the highest peak (average) stress occurred in the Lateral pillar for the healthy model. In contrast, for all other models, the highest stress was observed in the Central pillar. The stress index of the collapse area in the 3CS configuration ranged above 0.1. Specifically, it ranged from 0.073 to 0.123 in the normal screw-hole model and from 0.061 to 0.103 in the bone graft model.

The peak and average stress values for the health, sclerotic screw hole, and bone graft models are presented in Table 6. In both the sclerotic screw hole and bone graft models, the peak and average stresses in the overall model, collapse area, lateral pillar, central pillar, and medial pillar were significantly higher than those in the healthy model (Table 1). The bone graft model generally showed lower stress values compared to the sclerotic screw-hole model, except in the DHS+CS and FNS configurations, where the peak stress in the overall model increased. In the overall model, the DHS+CS configuration showed the highest peak and average stress values in both the sclerotic screw hole and bone graft models. In the sclerotic screw-hole models, the peak stress for DHS+CS was 56.75 MPa, and the average stress was 4.45 MPa. In the bone graft group, the peak and average stresses were 61.23 MPa and 14.6 MPa, respectively. In the collapse area, the 3CS configuration had the highest peak and average stress values in the sclerotic screw-hole models. The peak stress for 3CS was 17.54 MPa, and the average stress was 2.01 MPa. In the bone graft group, the highest peak stress was 14.6 MPa of the DHS+CS configuration, while the highest average stress was 1.83 MPa of the 3CS configuration. In the lateral, central, and medial pillars, the highest peak and average stress values were observed in the central pillar for both the sclerotic screw hole and bone graft models. Regarding the stress index in the collapse area, all screw-hole configurations exhibited values greater than 0.1 in both the sclerotic screw hole and bone graft groups.

**TABLE 6:** Stress in sclerotic screw hole and bone graft models.

Model	Peak (average) stress (Mpa)					Stress index of collapse area^b^
	Overall model	Collapse area^a^	Lateral pillar	Central pillar	Medial pillar	
3CS	39.57 (4.21)	**17.54 (2.01)**	8.5 (2)	**17.54 (2.49)**	8.16 (0.93)	**0.105–0.176**
DHS+CS	**56.75 (4.45)**	16.12 (1.97)	8.63 (1.94)	**16.12 (2.31)**	9.99 (1.19)	**0.096–0.162**
FNS	48.6 (4.37)	15 (1.96)	10.76 (1.96)	**15 (2.43)**	6.98 (0.85)	**0.089–0.151**
3CS (bone graft)	39.48 (4.17)	12.13 **(1.83)**	6.54 (1.93)	**12.13 (2.27)**	7.18 (0.84)	**0.072–0.122**
DHS+CS (bone graft)	**61.23 (4.36)**	**14.6** (1.79)	6.06 (1.9)	**14.6 (2.11)**	9.95 (1.08)	**0.087–0.147**
FNS (bone graft)	48.88 (4.33)	14.36 (1.8)	10.01 (1.94)	**14.39 (2.27)**	7.49 (0.76)	**0.086–0.144**

^a^ The collapse area is the collapsed necrotic region that typically occurs in the femoral head, involving the lateral, central, and medial pillars.

^b^ Stress index = effective stress/yield strength. Microfractures form in the collapse area when the stress index is >0.1. In the Overall model, the peak (average) stress was highest in the DHS+CS model, regardless of whether the sclerotic screw hole models or the bone graft models was used, with values of 56.75 (4.45) and 61.23 (4.36), respectively. In the Collapse area, the average stress was highest in the 3CS model for both the sclerotic screw hole models and the bone graft models, with values of 2.01 and 1.83, respectively. For the Collapse area peak stress, the highest value in the sclerotic screw hole models was observed in the 3CS model (17.54), whereas in the bone graft models, the highest peak stress occurred in the DHS+CS model, with a value of 14.6. For the three-column regions (Lateral, Central, and Medial pillars), the highest peak (average) stress occurred in the Central pillar across all models. In the Stress index of the collapse area, bold values indicate models where the stress index exceeds 0.1.

**FIGURE 2:**
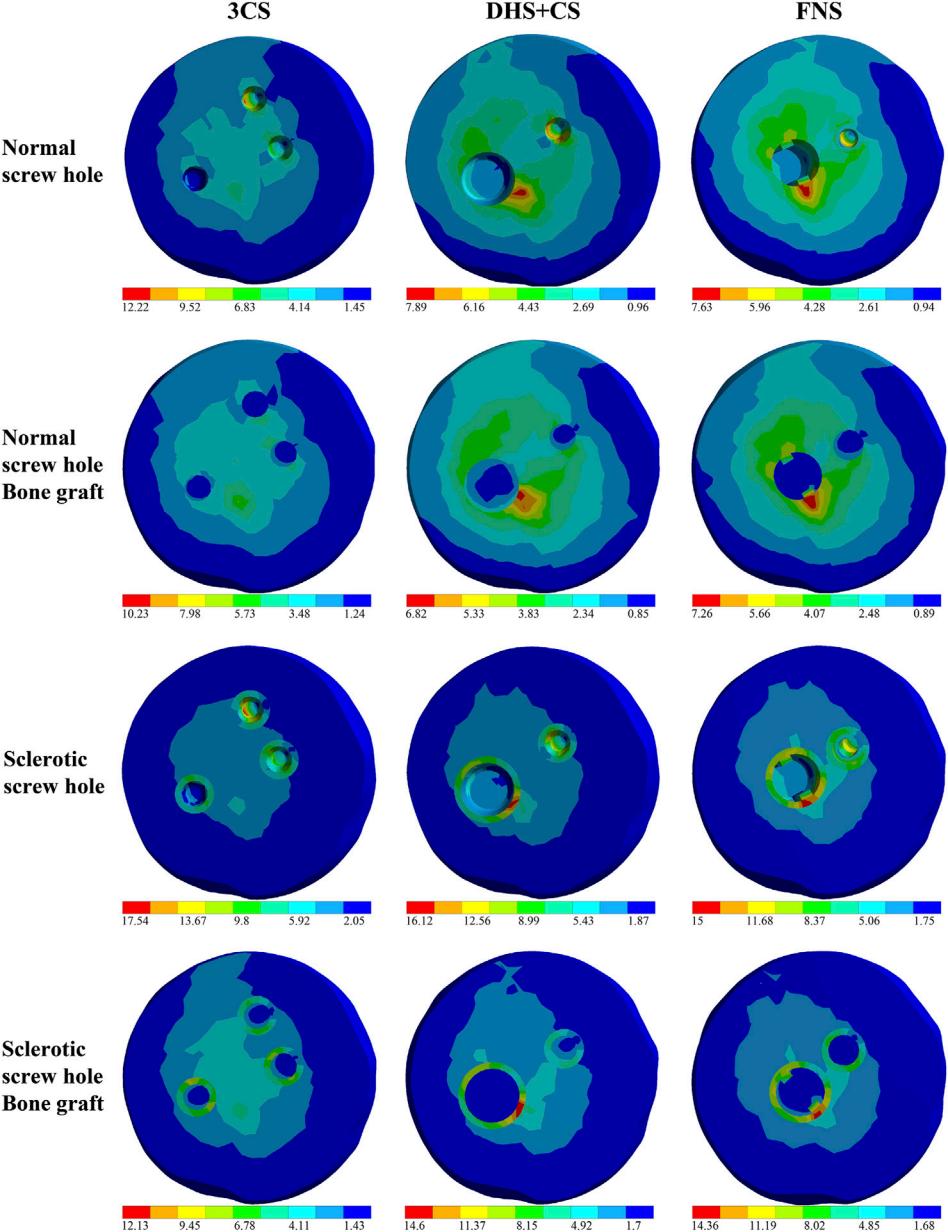
Stress distribution in the collapse area of the experiment models. Screw-hole models were represented as follows: 3CS (triple cannulated screws), DHS+CS (dynamic hip screws with cannulated screws), and FNS (femoral neck system). The red areas represent the stress intensive area.

Figure 1E shows the stress distribution in the collapse area of the healthy model in the control group, while Figure 2 illustrates the stress distribution in the collapse area for different screw-hole configurations in the experimental groups. The sclerotic screw-hole group exhibited the most concentrated stress, primarily around the screw holes, whereas the healthy model showed a more evenly distributed stress pattern. The red areas represent regions of high stress. Comparatively, in the healthy model, the red area was primarily concentrated in the central pillar. In the 3CS screw-hole configuration, the red area was mainly located at the deepest part of the uppermost CS screw hole. In the DHS+CS configuration, the red areas were concentrated in two places: the deepest part of the uppermost CS screw hole and around the DHS screw hole. For the FNS configuration, the red area was primarily around the screw hole of the dynamic rod, with no red stress concentration around the upper anti-rotation screw.

### 3.2 Evaluation of the strain range

Table 7 shows the strain ranges of the three pillars for the healthy, normal screw hole, and bone graft models. In general, the strain range across all screw hole configuration models, regardless of bone grafting, exceeded that of the healthy model. The bone graft models consistently exhibited lower strain ranges compared to the normal screw-hole models. In the normal screw-hole models, the 3CS screw-hole configuration resulted in the highest strain range in the central pillar, with a value of 4.18e-003. Similarly, in the bone graft models, the 3CS screw-hole configuration also displayed the largest strain range in the central pillar, with a value of 3.37e-003.

**TABLE 7:** Strain range of three pillars in health, normal screw hole, and bone graft models.

Model	Lateral pillar	Central pillar	Medial pillar
Healthy model	**2.93e-003**	2.03e-003	1.67e-003
3CS	2.81e-003	**4.18e-003**	2.49e-003
DHS+CS	3.08e-003	3.12e-003	3.14e-003
FNS	3.07e-003	3.45e-003	2.23e-003
3CS (bone graft)	2.81e-003	**3.37e-003**	2.21e-003
DHS+CS (bone graft)	2.92e-003	2.95e-003	3.06e-003
FNS (bone graft)	2.95e-003	3.06e-003	2.12e-003

In the Healthy model, the highest strain occurred in the Lateral pillar, with a value of 2.93e-003. In both the normal screw hole models and the bone graft models, the highest strain was observed in the Central pillar of the 3CS model, with values of 4.18e-003 and 3.37e-003, respectively. Strain range*: larger strain ranges, represented by the difference between the maximum and minimum strain values, correspond to fewer loading cycles and an increased likelihood of fatigue.

Table 8 provides the strain ranges for the sclerotic screw hole and bone graft models. The bone graft models generally showed reduced strain ranges when compared to the sclerotic screw-hole models. In the sclerotic screw-hole models, the 3CS screw-hole configuration had the largest strain range in the lateral pillar, measuring 3.14e-003. The same configuration in the bone graft models produced the highest strain range in the lateral pillar, with a value of 2.96e-003.

**TABLE 8:** Strain range of three pillars in sclerotic screw hole and bone graft models.

Model	Lateral pillar	Central pillar	Medial pillar
3CS	**3.14e-003**	2.31e-003	1.77e-003
DHS+CS	2.94e-003	2.36e-003	2.21e-003
FNS	2.91e-003	2.28e-003	1.68e-003
3CS (bone graft)	**2.96e-003**	2.29e-003	1.63e-003
DHS+CS (bone graft)	2.79e-003	2.35e-003	2.08e-003
FNS (bone graft)	2.75e-003	2.02e-003	1.68e-003

In both the sclerotic screw hole models and the bone graft models, the highest strain was observed in the Lateral pillar of the 3CS model, with values of 3.14e-003 and 2.96e-003, respectively. Strain range*: larger strain ranges, represented by the difference between the maximum and minimum strain values, correspond to fewer loading cycles and an increased likelihood of fatigue.

## 4 Discussion

As the fragile regional blood circulation, biomechanical environment, and anatomical characteristics of the femoral neck, ONFH and nonunion have emerged as the primary complication of femoral neck fracture (Dedrick et al., 1986; Karaeminogullari et al., 2004; Damany et al., 2005). Patients with femoral neck fractures undergoing internal fixation treatment still face a significant risk of developing ONFH within 1–2 years after the removal of internal fixation, even if the fracture has fully healed (Kang et al., 2016). According to a review study, the incidence of ONFH related to the removal of internal fixation is not uncommon (Jiang et al., 2023), which even reached 55.4% (Kim et al., 2022). Some scholars suppose that the occurrence of femoral head necrosis after femoral neck fracture must have biomechanical reasons, and the collapse is a direct consequence of the decreased mechanical performance of the necrotic femoral head. Kim et al. (1991) conducted mechanical testing on the subchondral bone and central cancellous bone of necrotic femoral heads, thereby establishing a direct relationship between the reduction in mechanical properties and the femoral head. Therefore, some researchers advocate that internal fixations cannot be removed (Chu et al., 2020), preferring retention of the implant after femoral neck fracture union to minimize the biomechanical changes (Ai et al., 2013; Li et al., 2018). However, the prolonged retention of the internal fixation implant following surgery for femoral neck fractures could result in osteosclerosis around the screw holes in the femoral head, potentially contributing to the development of ONFH (Liu et al., 2022b). Therefore, we conducted a finite element study considering various screw-hole configurations in the femoral head, along with osteosclerosis around the screw hole, following the union of femoral neck fractures, to explore the biomechanical behavior of the screw hole in the femoral head after the removal of the implant.

This study primarily focuses on analyzing the peak stress, stress index, and strain range in the collapsed region of the femoral head. Evidence suggests that peak stress is a reliable biomechanical marker for assessing the risk of femoral head collapse (Li et al., 2019). According to the classification of three pillars, early-stage femoral head collapse in ONFH may result from microfractures that occur when local von Mises stress exceeds the bone’s yield strength. Following the removal of internal fixation, tensile, compressive, and shear forces concentrate on the original fracture site, disrupting the biomechanical balance of the femoral neck and leading to trabecular adjustments, which increase the risk of microfractures or re-fractures (Shah et al., 2015). The stress index, defined as the ratio of effective stress to yield strength, indicates that bone theoretically yields or fractures when this index exceeds 0.1 (Yang et al., 2002; Wen et al., 2020). Bayraktar et al. reported that the yield strength of femoral bone is 133.6 ± 34.1 MPa, corresponding to a critical stress range of 99.5–167.7 MPa (Bayraktar et al., 2004). The bone fatigue process is characterized by progressive damage, with the total strain range determining the number of cycles required to reach fatigue failure. A negative correlation exists between the total strain range and the number of loading cycles necessary to induce fatigue fractures—larger strain ranges correspond to fewer loading cycles, thereby increasing the likelihood of fatigue (Carter et al., 1981).

According to our results, although the DHS+CS screw-hole configuration showed the highest overall stress in the model, the 3CS configuration exhibited the highest stress, specifically in the collapse area. The 3CS configuration also produced the largest strain range, which was observed in the central pillar of the normal screw-hole models and in the lateral pillar of the sclerotic screw-hole models. In contrast, the FNS screw-hole configuration showed the lowest stress in most indicators, except for stress in the collapse area after bone grafting, where it was not the lowest among the three configurations. Therefore, the FNS screw-hole demonstrates a relatively lower mechanical risk of femoral head collapse. In the three-pillar classification of ONFH, the lateral pillar is the primary biomechanical support of the femoral head and a critical region for predicting collapse (Wen et al., 2020). A possible explanation for our findings is that the 3CS configuration caused two defects in the lateral pillar, whereas the DHS+CS and FNS configurations each resulted in only one defect. Additionally, the anti-rotation screw in the FNS configuration, with its smaller diameter and angled insertion compared to the larger cannulated screw in the DHS + CS configuration, causes relatively less damage to the lateral pillar. The reason why the highest stress occurs in the lateral pillar, while the largest strain range is found in the central pillar for the normal screw hole and bone graft models, yet shifts to the lateral pillar in the sclerotic screw hole and bone graft models, may be explained by findings from Wen et al. Their research indicates that necrosis in the central pillar significantly affects the mechanical behavior of both the lateral and medial pillars, potentially leading to altered stress and strain distribution across these regions (Wen et al., 2017). Additionally, due to the significant role of the proximal femur in human motion and its weight-bearing capability, it undergoes compression, tension, and torsional stresses. Under external forces, structures such as tension trabeculae, compression trabeculae, and the femoral calcar are formed in the proximal femur (Skedros and Brand, 2011). When internal fixation is implanted, some trabeculae are damaged, and after removal, the screw holes remain, with damaged compression trabeculae failing to fully recover. This leads to stress concentration in the deficient areas, particularly at the central pillar. In all screw-hole configurations, the stress level and strain range on the collapse area exceeds that in the healthy model, increasing the risk of femoral head collapse post-implant removal. This finding is consistent with the theory of micro-fracture proposed by Wang T. et al. (2014). Furthermore, a previous finite element analysis also confirmed that the removal of all inverted 3CS, following femoral neck fractures union, will result in the highest concentration of von Mises stress on the femoral head, surpassing the scenario where the implant is retained or the lowest femoral calcar screw is removed first. The improper removal of screws and the resulting load on the femur may indeed be closely associated with the occurrence of ONFH or the recurrence of femoral neck fractures (Wu et al., 2022).

Based on our study results, the sclerotic screw-hole configuration significantly increased stress values compared to the normal screw-hole configuration, while the strain range in the sclerotic screw holes was smaller than that in the normal screw holes, primarily due to the higher elastic modulus of the sclerotic bone. However, this does not imply that sclerotic screw-hole configurations are less prone to collapse clinically. Sclerotic bone forms as a result of long-term cyclic extreme stress, leading to trabecular necrosis and collapse around the screw hole (Liu et al., 2022b). When internal fixation devices are retained, biomechanical stresses primarily concentrate on the internal fixation materials. This increased stress loading induces changes in bone mass and microstructure, resulting in an increased bone volume fraction, quantity of trabeculae, and trabecular diameter (Wang et al., 2012). The normal femoral head is primarily composed of trabecular bone, which possesses a certain degree of elasticity (Goda et al., 2012). This sclerotic bone disrupts the femoral head’s normal elasticity, contributing to trabecular damage and increased bone mass from disuse, often signaling the irreversible progression of femoral head necrosis (Ting et al., 2016; Li et al., 2018). Additionally, the process of bone resorption takes only 3 weeks to alter the trabecular structure, while the process of bone formation requires 3 months to construct new bone with good mechanical properties (Wang C. et al., 2014). The dense plate-like trabeculae formed by sclerosis around the screw hole may hinder the revascularization of the original screw track, which could be one of the reasons why bone reconstruction cannot occur in the screw hole even after implant removal (Liu et al., 2022a).

In our study, the bone graft model showed a decrease in both stress values and strain range compared to those in the pre-grafting conditions. This indicates that bone grafting may reduce the risk of femoral head collapse post-implant removal as the von Mises stress on the weight-bearing region of the femoral head was predominantly borne by the grafted bone rather than the screw hole. Furthermore, grafting cancellous bone within the screw hole can enhance the biomechanical performance of the femoral neck by promoting stress distribution within the weight-bearing region of the bone, thereby mitigating the localized stress concentration and preventing subsequent femoral head collapse. Rosson et al. (1991) pointed out that in young adults, the bone volume at the screw hole is close to normal at 18 weeks after removing the steel plate, and they recommended avoiding full weight-bearing within 4 months after implant removal. Burstein et al. (1972) found that woven bone-filling screw holes could eliminate stress concentration effects around the screw holes within approximately 4 weeks. However, there is still limited research on how to facilitate the rapid restoration of full weight-bearing activity in patients after implant removal, especially concerning the improvement of stress distribution along the screw holes. We hypothesize that bone grafting for the screw hole after implant removal could alleviate von Mises stress concentration on the femoral head without the constraint of full weight-bearing. In response to this phenomenon, the authors suggest that before sclerotic bone forms around the internal fixation, timely removal of the screws and insertion of a new type of high-strength, biodegradable implant with a porous structure that promotes osteogenesis and vascularization could prevent the development of sclerotic bone, thereby halting femoral head necrosis. Alternatively, the development of a biomaterial that mimics the strength and biological function of femoral bone could be used to treat femoral neck fractures, helping in avoiding stress concentration around the implant and reducing the risk of femoral head necrosis.

There are still some limitations in our study. First, it does not account for the effects of surrounding muscles and ligaments on the femur, nor does it consider the potential variations in bone absorption associated with the three internal fixation methods. Second, the mechanical conduction mechanisms involved in femoral head collapse after the removal of internal fixation, following femoral neck fracture, are indeed complex, and our understanding of the exact underlying mechanisms remains incomplete. Third, regarding the changes in the von Mises stress location after bone grafting, we assume that the grafted bone alters the stress distribution, but we still need further studies to explore the mechanics. Additionally, the material properties of grafting bone were defined as normal cancellous. In clinical practice, it is difficult to maintain normal cancellous density in the grafted bone due to the uncontrollable material properties and qualities of the bone graft donor. Although our study provides valuable insights into the biomechanical behavior of screw holes in the femoral head under different conditions, the biomechanical test and the additional clinical trials involving larger patient populations and longer follow-up periods are needed, and we will perform in future studies.

## 5 Conclusion

The FNS screw hole presents a comparatively reduced mechanical risk of femoral head collapse, which may be attributed to the smaller defects it induces in the lateral pillar. On the other hand, sclerotic screw holes are associated with a higher risk of collapse, whereas bone grafting shows potential in improving biomechanical behavior, possibly decreasing the likelihood of femoral head collapse. This study provides a theoretical biomechanical basis for hip preservation strategies and informs the design of new internal fixation techniques and materials.

## Data Availability

The original contributions presented in the study are included in the article/supplementary material; further inquiries can be directed to the corresponding authors.

## Abbreviations

ONFH, osteonecrosis of the femoral head; OS, osteosclerosis; OP, osteoporosis; FE, finite element; FNF, femoral neck fracture; DHS, dynamic hip screw; CS, cannulated screws; CT, computed tomography; FNS, femoral neck system.

## References

[B1] Abrahamsen B. Van Staa T. Ariely R. Olson M. Cooper C. (2009). Excess mortality following hip fracture: a systematic epidemiological review. Osteoporos. Int. 20, 1633–1650. 10.1007/s00198-009-0920-3 19421703

[B2] Ai Z. S. Gao Y. S. Sun Y. Liu Y. Zhang C. Q. Jiang C. H. (2013). Logistic regression analysis of factors associated with avascular necrosis of the femoral head following femoral neck fractures in middle-aged and elderly patients. J. Orthop. Sci. 18, 271–276. 10.1007/s00776-012-0331-8 23114858

[B3] Alford J. W. Bradley M. P. Fadale P. D. Crisco J. J. Moore D. C. Ehrlich M. G. (2007). Resorbable fillers reduce stress risers from empty screw holes. J. Trauma 63, 647–654. 10.1097/01.ta.0000221042.09862.ae 18073615

[B4] Bayraktar H. H. Morgan E. F. Niebur G. L. Morris G. E. Wong E. K. Keaveny T. M. (2004). Comparison of the elastic and yield properties of human femoral trabecular and cortical bone tissue. J. Biomech. 37, 27–35. 10.1016/s0021-9290(03)00257-4 14672565

[B5] Bhandari M. Swiontkowski M. (2017). Management of acute hip fracture. N. Engl. J. Med. 377, 2053–2062. 10.1056/nejmcp1611090 29166235

[B6] Burstein A. H. Currey J. Frankel V. H. Heiple K. G. Lunseth P. Vessely J. C. (1972). Bone strength. The effect of screw holes. J. Bone Jt. Surg. Am. 54, 1143–1156. 10.2106/00004623-197254060-00001 4652047

[B7] Carter D. R. Caler W. E. Spengler D. M. Frankel V. H. (1981). Fatigue behavior of adult cortical bone: the influence of mean strain and strain range. Acta Orthop. Scand. 52, 481–490. 10.3109/17453678108992136 7331784

[B8] Chen J. Ma J. X. Wang Y. Bai H. H. Sun L. Wang Y. (2019). Finite element analysis of two cephalomedullary nails in treatment of elderly reverse obliquity intertrochanteric fractures: zimmer natural nail and proximal femoral nail antirotation-ΙΙ. J. Orthop. Surg. Res. 14, 422. 10.1186/s13018-019-1468-3 31823801 PMC6902592

[B9] Chu K. Zhang X. Lu X. Chen X. (2020). Risk of micro-fracture in femoral head after removal of cannulated screws for femoral neck fracture. Zhongguo Xiu Fu Chong Jian Wai Ke Za Zhi 34, 1091–1095. 10.7507/1002-1892.202001076 32929899 PMC8171717

[B10] Cooke T. D. V. Sled E. A. (2009). Optimizing limb position for measuring knee anatomical axis alignment from standing knee radiographs. J. Rheumatology 36, 472–477. 10.3899/jrheum.080732 19286859

[B11] Damany D. S. Parker M. J. Chojnowski A. (2005). Complications after intracapsular hip fractures in young adults. A meta-analysis of 18 published studies involving 564 fractures. Injury 36, 131–141. 10.1016/s0020-1383(04)00175-5 15589931

[B12] Dedrick D. K. Mackenzie J. R. Burney R. E. (1986). Complications of femoral neck fracture in young adults. J. Trauma 26, 932–937. 10.1097/00005373-198610000-00013 3773004

[B13] Fan Z. Huang Y. Su H. Jiang T. (2021). How to choose the suitable FNS specification in young patients with femoral neck fracture: a finite element analysis. Injury 52, 2116–2125. 10.1016/j.injury.2021.05.043 34154816

[B14] Fu L. Zhao H.-W. Zhu Y.-X. Zou Q. Liu C.-S. Zhao B. (2012). “Biomechanics analysis of human proximal femur under four different standing postures based on finite element method,” in 2012 IEEE Symposium on Robotics and Applications ISRA, Kuala Lumpur, Malaysia, 03-05 June 2012, 122–124. 10.1109/isra.2012.6219136

[B15] Goda I. Assidi M. Ganghoffer J. F. (2012). Cosserat 3D anisotropic models of trabecular bone from the homogenisation of the trabecular structure. Comput. Methods Biomech. Biomed. Engin 15 (Suppl. 1), 288–290. 10.1080/10255842.2012.713645 23009512

[B16] Goudie E. B. Duckworth A. D. White T. O. (2018). Femoral neck fractures in the young. in Proximal Femur Fractures: An evidence-based approach to evaluation and management. Springer, 47–58. 10.1007/978-3-319-64904-7_5

[B17] Hanson B. Van Der Werken C. Stengel D. (2008). Surgeons' beliefs and perceptions about removal of orthopaedic implants. BMC Musculoskelet. Disord. 9, 73. 10.1186/1471-2474-9-73 18501014 PMC2430567

[B18] Hou Y. Zhu X. (2006). Investigation in the dependency of stiffness of cancellous bone on apparent density--based on the combination model of rod-rod structure and perforated plate structure. Sheng Wu Yi Xue Gong Cheng Xue Za Zhi 23, 78–81. 16532815

[B19] Huang H. K. Su Y. P. Chen C. M. Chiu F. Y. Liu C. L. (2010). Displaced femoral neck fractures in young adults treated with closed reduction and internal fixation. Orthopedics 33, 873. 10.3928/01477447-20101021-15 21162504

[B20] Jiang Q. Deng Y. Liu Y. Zhao Z. Chen Y. Bai X. (2023). Association of hardware removal with secondary osteonecrosis following femoral neck fractures: a systematic review and meta-analysis. J. Orthop. Surg. Res. 18, 931. 10.1186/s13018-023-04427-8 38057793 PMC10702042

[B21] Jian-Qiao Peng M. Chen H. Y. Ju X. Hu Y. Ayoub A. Khambay B. (2020). Comparative analysis for five fixations of Pauwels-I by the biomechanical finite-element method. J. Invest. Surg. 33, 428–437. 10.1080/08941939.2018.1533054 30516078

[B22] Kang J. S. Moon K. H. Shin J. S. Shin E. H. Ahn C. H. Choi G. H. (2016). Clinical results of internal fixation of subcapital femoral neck fractures. Clin. Orthop. Surg. 8, 146–152. 10.4055/cios.2016.8.2.146 27247738 PMC4870316

[B23] Karaeminogullari O. Demirors H. Atabek M. Tuncay C. Tandogan R. Ozalay M. (2004). Avascular necrosis and nonunion after osteosynthesis of femoral neck fractures: effect of fracture displacement and time to surgery. Adv. Ther. 21, 335–342. 10.1007/bf02850038 15727403

[B24] Kim C. H. Shin M. Lee D. Choi S. J. Moon D. H. (2022). Hidden osteonecrosis of the femoral head after healed femoral neck fractures: magnetic resonance imaging study of 58 consecutive patients. Arch. Orthop. Trauma Surg. 142, 1443–1450. 10.1007/s00402-021-03802-6 33611613

[B25] Kim Y. M. Lee S. H. Lee F. Y. Koo K. H. Cho K. H. (1991). Morphologic and biomechanical study of avascular necrosis of the femoral head. Orthopedics 14, 1111–1116. 10.3928/0147-7447-19911001-10 1946047

[B26] Li J. Wang M. Zhou J. Han L. Zhang H. Li C. (2018). Optimum configuration of cannulated compression screws for the fixation of unstable femoral neck fractures: finite element analysis evaluation. Biomed. Res. Int. 2018, 1–10. 10.1155/2018/1271762 PMC6304632 30627534

[B27] Li T. X. Huang Z. Q. Li Y. Xue Z. P. Sun J. G. Gao H. H. (2019). Prediction of collapse using patient-specific finite element analysis of osteonecrosis of the femoral head. Orthop. Surg. 11, 794–800. 10.1111/os.12520 31663283 PMC6819171

[B28] Liu Y. Liang H. Zhou X. Song W. Shao H. He Y. (2022a). Micro-computed tomography analysis of femoral head necrosis after long-term internal fixation for femoral neck fracture. Orthop. Surg. 14, 1186–1192. 10.1111/os.13318 35587534 PMC9163795

[B29] Liu Y. Song W. Liang H. Li C. Niu W. Shao H. (2022b). Comparison of femoral mechanics before and after internal fixation removal and the effect of sclerosis on femoral stress: a finite element analysis. BMC Musculoskelet. Disord. 23, 930. 10.1186/s12891-022-05888-4 36271382 PMC9587577

[B30] Mundi S. Pindiprolu B. Simunovic N. Bhandari M. (2014). Similar mortality rates in hip fracture patients over the past 31 years. Acta Orthop. 85, 54–59. 10.3109/17453674.2013.878831 24397744 PMC3940992

[B31] Niemann M. Maleitzke T. Jahn M. Salmoukas K. Braun K. F. Graef F. (2023). Restoration of hip geometry after femoral neck fracture: a comparison of the femoral neck system (FNS) and the dynamic hip screw (DHS). Life (Basel) 13, 2073. 10.3390/life13102073 37895454 PMC10608621

[B32] Oefner C. Herrmann S. Kebbach M. Lange H. E. Kluess D. Woiczinski M. (2021). Reporting checklist for verification and validation of finite element analysis in orthopedic and trauma biomechanics. Med. Eng. Phys. 92, 25–32. 10.1016/j.medengphy.2021.03.011 34167708

[B33] Papini M. Zdero R. Schemitsch E. H. Zalzal P. (2007). The biomechanics of human femurs in axial and torsional loading: comparison of finite element analysis, human cadaveric femurs, and synthetic femurs. J. Biomech. Eng. 129, 12–19. 10.1115/1.2401178 17227093

[B34] Rosson J. Murphy W. Tonge C. Shearer J. (1991). Healing of residual screw holes after plate removal. Injury 22, 383–384. 10.1016/0020-1383(91)90100-s 1806500

[B35] San Antonio T. Ciaccia M. Müller-Karger C. Casanova E. (2012). Orientation of orthotropic material properties in a femur FE model: a method based on the principal stresses directions. Med. Eng. Phys. 34, 914–919. 10.1016/j.medengphy.2011.10.008 22100056

[B36] Shah S. N. Kapoor C. S. Jhaveri M. R. Golwala P. P. Patel S. (2015). Analysis of outcome of avascular necrosis of femoral head treated by core decompression and bone grafting. J. Clin. Orthop. Trauma 6, 160–166. 10.1016/j.jcot.2015.03.008 26155051 PMC4488021

[B37] Skedros J. G. Brand R. A. (2011). Biographical sketch: Georg Hermann von Meyer (1815-1892). Clin. Orthop. Relat. Res. 469, 3072–3076. 10.1007/s11999-011-2040-6 21901583 PMC3183195

[B38] Szymski D. Walter N. Melsheimer O. Grimberg A. Alt V. Steinbruck A. (2023). Mortality after hemiarthroplasty for femoral neck fractures. Dtsch. Arztebl Int. 120, 297–298. 10.3238/arztebl.m2023.0007 37482793 PMC10391523

[B39] Ting B. L. Heng M. Vrahas M. S. Rodriguez E. K. Harris M. B. Weaver M. J. (2016). Is disuse osteopenia a favorable prognostic sign after femoral neck fracture? J. Orthop. Trauma 30, 496–502. 10.1097/bot.0000000000000635 27243346

[B40] Wang C. Wang X. Xu X. L. Yuan X. L. Gou W. L. Wang A. Y. (2014a). Bone microstructure and regional distribution of osteoblast and osteoclast activity in the osteonecrotic femoral head. PLoS One 9, e96361. 10.1371/journal.pone.0096361 24800992 PMC4011745

[B41] Wang H. Ji B. Liu X. S. Guo X. E. Huang Y. Hwang K. C. (2012). Analysis of microstructural and mechanical alterations of trabecular bone in a simulated three-dimensional remodeling process. J. Biomech. 45, 2417–2425. 10.1016/j.jbiomech.2012.06.024 22867764

[B42] Wang K. Ni M. Liao P. Dou B. Yan X. Lv L. (2021). Fracture morphology and biomechanical characteristics of Pauwels III femoral neck fractures in young adults. Injury 52, 3227–3238. 10.1016/j.injury.2021.08.025 34481668

[B43] Wang M. Wang S. Duan C. (2017). “Finite element analysis of femoral mechanical properties in MATLAB environment,” in 2017 IEEE International Conference on Cybernetics and Intelligent Systems (CIS) and IEEE Conference on Robotics, Ningbo, China, 19-21 November 2017 (Ningbo, China: Automation and Mechatronics RAM), 535–538.

[B44] Wang T. Sun J. Y. Zha G. C. Jiang T. You Z. J. Yuan D. J. (2014b). Analysis of risk factors for femoral head necrosis after internal fixation in femoral neck fractures. Orthopedics 37, e1117–e1123. 10.3928/01477447-20141124-60 25437087

[B45] Wang Y. Ma J. X. Yin T. Han Z. Cui S. S. Liu Z. P. (2019). Correlation between reduction quality of femoral neck fracture and femoral head necrosis based on biomechanics. Orthop. Surg. 11, 318–324. 10.1111/os.12458 31025811 PMC6594541

[B46] Wen P. Zhang Y. Hao L. Yue J. Wang J. Wang T. (2020). The effect of the necrotic area on the biomechanics of the femoral head - a finite element study. BMC Musculoskelet. Disord. 21, 211. 10.1186/s12891-020-03242-0 32252708 PMC7137335

[B47] Wen P. F. Guo W. S. Zhang Q. D. Gao F. Q. Yue J. A. Liu Z. H. (2017). Significance of lateral pillar in osteonecrosis of femoral head: a finite element analysis. Chin. Med. J. Engl. 130, 2569–2574. 10.4103/0366-6999.217077 29067956 PMC5678256

[B48] Wu L. Sun J. Fang N. Peng Q. Gao S. Liu L. (2022). Should cannulated screws be removed after a femoral neck fracture has healed, and how? A finite element analysis of the femur before and after cannulated screw removal. Int. Orthop. 46, 2393–2403. 10.1007/s00264-022-05516-9 35852654

[B49] Xiao D. Ye M. Li X. Yang L. (2015). Development of femoral head interior supporting device and 3D finite element analysis of its application in the treatment of femoral head avascular necrosis. Med. Sci. Monit. 21, 1520–1526. 10.12659/msm.893354 26010078 PMC4456983

[B50] Yang J. J. Lin L. C. Chao K. H. Chuang S. Y. Wu C. C. Yeh T. T. (2013). Risk factors for nonunion in patients with intracapsular femoral neck fractures treated with three cannulated screws placed in either a triangle or an inverted triangle configuration. J. Bone Jt. Surg. Am. 95, 61–69. 10.2106/jbjs.k.01081 23283374

[B51] Yang J. W. Koo K. H. Lee M. C. Yang P. Noh M. D. Kim S. Y. (2002). Mechanics of femoral head osteonecrosis using three-dimensional finite element method. Arch. Orthop. Trauma Surg. 122, 88–92. 10.1007/s004020100324 11880908

[B52] Yosibash Z. Trabelsi N. Milgrom C. (2007). Reliable simulations of the human proximal femur by high-order finite element analysis validated by experimental observations. J. Biomech. 40, 3688–3699. 10.1016/j.jbiomech.2007.06.017 17706228

[B53] Zhan S. Jiang D. Hu Q. Wang M. Feng C. Jia W. (2024). Single-plane osteotomy model is inaccurate for evaluating the optimal strategy in treating vertical femoral neck fractures: a finite element analysis. Comput. Methods Programs Biomed. 245, 108036. 10.1016/j.cmpb.2024.108036 38244341

[B54] Zhang L. Zhu B. Chen L. Wang W. Zhang X. Zhang J. (2023). The impact of coronal configuration of the proximal femur on its mechanical properties and the validation of a new theoretical model: finite element analysis and biomechanical examination. Orthop. Surg. 15, 62–69. 10.1111/os.13537 36250538 PMC9837247

[B55] Zhou G. Zhang Y. Zeng L. He W. Pang Z. Chen X. (2015). Should thorough Debridement be used in Fibular Allograft with impaction bone grafting to treat Femoral Head Necrosis: a biomechanical evaluation. BMC Musculoskelet. Disord. 16, 140. 10.1186/s12891-015-0593-3 26059456 PMC4460632

[B56] Zhou S. Jung S. Hwang J. (2019). Mechanical analysis of femoral stress-riser fractures. Clin. Biomech. (Bristol, Avon) 63, 10–15. 10.1016/j.clinbiomech.2019.02.004 30784785

[B57] Zielinski S. M. Heetveld M. J. Bhandari M. Patka P. Van Lieshout E. M. (2015). Implant removal after internal fixation of a femoral neck fracture: effects on physical functioning. J. Orthop. Trauma 29, e285–e292. 10.1097/bot.0000000000000358 26165264

